# The effects of medium cut-off and high-flux membranes on activated clotting time of patients on hemodialysis

**DOI:** 10.3389/fneph.2023.1133910

**Published:** 2023-02-14

**Authors:** Isabela Pereira Lucca, Rachel Armani, Aluizio B. Carvalho, Silvia R. Manfredi, Monique V. Rocha E. Silva, Thamires B. Gratão, Lidia Silva, Renato Watanabe, Maria Eugenia Canziani

**Affiliations:** ^1^ Department of Medicine, Paulista School of Medicine, Federal University of São Paulo, São Paulo, São Paulo, Brazil; ^2^ Hrim - Kidney Hospital, São Paulo, São Paulo, Brazil

**Keywords:** medium cut-off membrane, Theranova, high-flux dialysis, coagulation pattern, activated clotting time

## Abstract

**Introduction:**

The interaction between blood and dialysis membrane increases the risk of clot formation. Membrane properties can interfere with coagulation activation during dialysis. Heparin is usually used to ensure anticoagulation, which can be monitored by the Activated Clotting Time (ACT) test. The purpose of this study was to compare the ACT of patients with chronic kidney disease (CKD) undergoing hemodialysis with high-flux (HF) and medium cut-off (MCO) membranes.

**Methods:**

This is a prospective, randomized, crossover study in which 32 CKD patients were dialyzed for 12 weeks with each membrane. Blood clotting measured by ACT was evaluated at the beginning, 2nd, and 4th hour of the dialysis session. Throughout the study, there were no changes in the dose or administration method of heparin.

**Results:**

Patients mainly were middle-aged, non-black males on hemodialysis for eight years. Before randomization, ACT values were 132 ± 56, 195 ± 60, and 128 ± 32 seconds at pre-heparinization, 2nd and 4th hour, respectively. After 12 weeks, ACT values in HF and MCO groups were 129 ± 17, 205 ± 65 and 139 ± 38 seconds, and 143 ± 54, 219 ± 68 and 142 ± 45 seconds, respectively. An ANOVA model adjusted and unadjusted for repeated measures showed a significant time but no treatment or interaction effects. In an additional paired-sample analysis, no difference between ACT values of HF and MCO Groups was observed.

**Discussion and Conclusion:**

There was no difference regarding the ACT test during dialysis therapy using HF or MCO membranes. This data suggests that no adjustment in the dose or administration method of heparin is necessary with the use of MCO dialysis membranes.

## Introduction

The number of patients undergoing hemodialysis (HD) is continually increasing worldwide (1). Although substantial technological improvements have been made in HD treatment in the last decades, this population’s hospitalization and mortality rates are still very high (2). These data indicate that there is room to enhance the HD technique.

New dialysis membranes have been developed to accomplish better outcomes in hemodialysis patients. However, there is a lack of information regarding the safety profile of anticoagulation during dialysis procedures using these new membranes. Of note, if patients with CKD have a higher risk of bleeding mainly due to platelet dysfunction, on the other hand, they are chronically exposed to extracorporeal circuits, which favors hypercoagulability (3, 4). Unfractionated heparin is the medication usually administered to ensure anticoagulation during the dialysis procedure (5). The narrow therapeutic window for performing adequate anticoagulation without leading to bleeding emphasizes the need to monitor anticoagulation during hemodialysis (6). The adequacy of heparinization can be assessed with the Activated Clotting Time (ACT) test. This test is used to determine heparin’s pharmacokinetics and pharmacodynamics properties, showing a good correlation with heparin concentrations during the hemodialysis session (7). It is essential to mention that ACT is more practical than the gold-standard test for assessing heparin activity (activated partial thromboplastin time or APTT). ACT is considered a point-of-care test to monitor anticoagulation in HD, as it is quick, reproducible, and possible to perform in the dialysis room (8).

The specific role of dialysis membranes on blood coagulation has been of concern since different membrane biocompatibility properties can interfere with the coagulation pattern (9–11). It is well known that exposure to the dialyzer leads to platelet and complement activation. These activations consequently stimulate the coagulation cascade, which starts right after the beginning of the dialysis session (12–15). Recently, new medium cut-off membranes (MCO) were developed to optimize the removal of uremic toxins, and no information regarding its effect on the coagulation pattern is available (16). The purpose of this study was to compare the ACT of patients with chronic kidney disease (CKD) undergoing hemodialysis with MCO and high-flux (HF) membranes.

## Materials and methods

### Study population

This trial is a pre-defined exploratory analysis of a prospective study that compared the effect of MCO and HF hemodialysis membranes in CKD patients. This study included 32 adult patients with adequate vascular access, undergoing hemodialysis for at least six months. The exclusion criteria were inadequate dialysis (as defined by KtV < 1.2), the presence of heart failure (III or IV New York Heart Association class), peripheral arterial disease, stroke, active inflammation or infectious disease within three months before the enrollment, as well as pregnancy or breastfeeding.

The study was approved by the Ethics Committee of the Federal University of São Paulo (n. 026386/2019) and registered in the Brazilian Registry of Clinical Trials (REBEC, RBR-2m67zd). All patients signed the informed consent form as the first procedure.

### Study design

**Figure 1:**
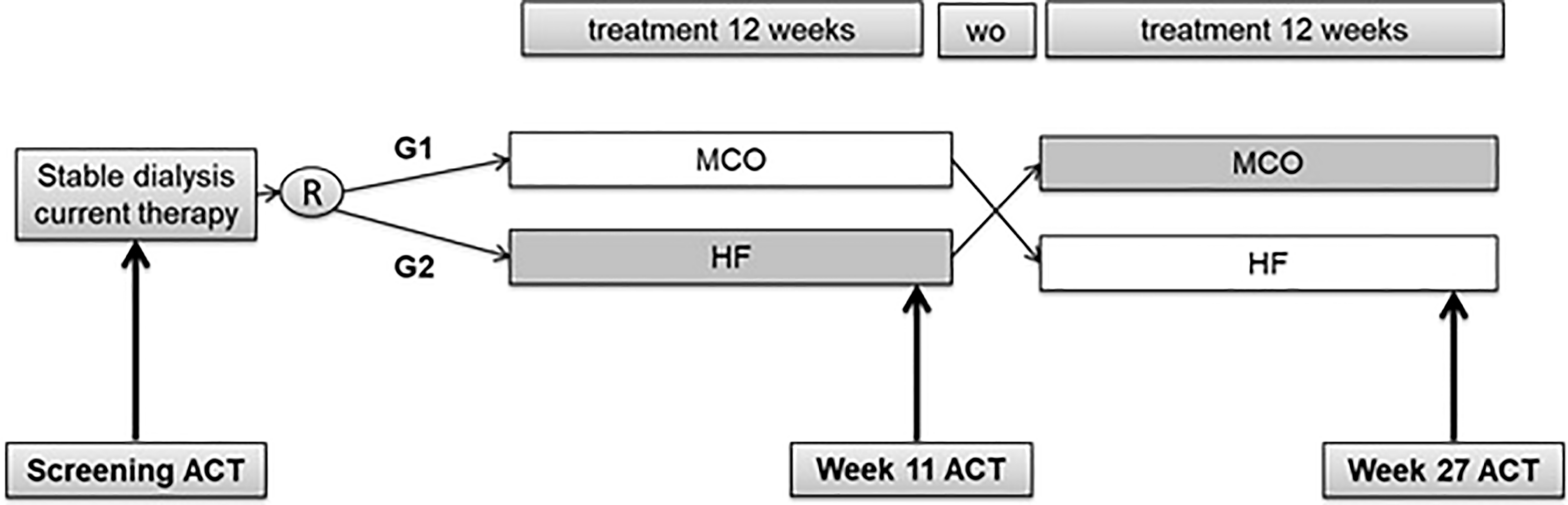
Study design. ACT, Activated Clotting Time; R, Randomization; WO, washout; MCO, medium cut-off membrane; HF, High Flux membrane; G1, Group 1 (MCO followed by HF); G2, Group 2 (HF followed by MCO).

This study is a randomized, crossover, open-label, 28-week follow-up clinical trial. Selected patients were randomized in a 1:1 ratio to 12-week treatment with a single use of MCO (Theranova 400 dialyzer, Baxter Healthcare, USA) or high-flux (FX 100, Fresenius, Germany) dialysis membranes. After the first treatment period, patients underwent a 4-week washout with reusable HF dialyzers (Hemoflow, Fresenius, Germany) and switched to the second 12-week period, as shown in **Figure 1**. Hemodialysis sessions during treatments and washout were performed for 4 hours, three times a week, with blood and dialysate flows of 300 and 600mL/min, respectively.

All patients were submitted to an ACT test to construct 3-point curves at the screening week and the end of each treatment (Week 11 and Week 27). The ACT tests (reference values: 80 to 140 seconds) were performed in the ACT-500 equipment (Fundação Adib Jatene, São Paulo, Brazil), comprising a sensor that indicates the time, in seconds, required for the blood sample coagulation. In this test, 2mL blood samples were collected from the arterial pathway of the vascular access in a specific tube, and the same operator performed all tests. In addition, the dialysis room temperature was checked on the days of the ACT tests.

Heparinization was carried out in single bolus or fractionated into 60 and 40% at the beginning (pre-heparinization) and 2^nd^ hour of dialysis, respectively. The unfractionated porcine sodium heparin (Blau Pharmaceutical, São Paulo, Brazil) was used for all patients. An adequate ACT test curve consists of values ranging from 80 to 180% above the baseline after 2 hours of heparinization, and 40% above the baseline, after 4 hours (17). One week before randomization, ACT was performed, and for those whose coagulation pattern was not adequate, the heparin dose was adjusted, and another ACT curve was constructed. Throughout the study, there were no changes in the dose or administration method of heparinization defined during the screening week. Also, bleeding events and extracorporeal system clotting were carefully evaluated and recorded throughout the study.

At baseline, demographic and clinical data were reported, including using medications such as erythropoietin and acetylsalicylic acid. In addition, blood samples were collected in a fasting state at baseline and the end of each treatment period. Besides routine chemistry and hematology tests, inflammatory markers such as IL-6 [reference range (RR) 1.5 to 7.0 pg/mL, Merck Diagnostics’ kit, Germany], IL-1β (RR 0.5 to 12.0 pg/mL, Merck Diagnostics’ kit, Germany), TNFα (RR 1.2 to 15.3 pg/mL, Merck Diagnostics’ kit, Germany), CRP (RR lower than 1.0 mg/dL) and von Willebrand factor (vWf, RR 78 to 1030 ng/mL, Elabscience’s kit, USA) were measured.

### Statistical analysis

Mean, standard deviation or frequencies were calculated, and the Kolmogorov-Smirnov test was used to investigate the distribution of all variables. Patients were initially grouped according to randomization in Group 1, composed of patients who started the study with MCO, followed by HF membrane, and Group 2, by those who started with HF, followed by MCO. Comparisons of continuous variables between groups were performed using Student’s t-test or the Mann-Whitney U-test and comparisons of proportions using chi-squared analysis or Fisher’s exact test, as appropriate. Due to the study’s crossover design, the interferences of the order of treatments (sequence effect) and the effect of the first treatment on the second one (carryover effect) were evaluated. In the absence of those conditions, patients were regrouped according to the treatment (MCO and HF), regardless of the observation moment. As appropriate, comparison within groups was evaluated by paired-sample t-test or Wilcoxon. ANOVA analysis for repeated measures evaluating time, group, and interaction effects was performed with and without covariables to compare MCO and High-Flux groups. The selected covariables were the use of erythropoietin, acetylsalicylic acid dose, hemoglobin, platelets, CRP, TNFα, IL-6, IL-1β, and vWf. P < 0.05 was considered statistically significant. All statistical analysis was performed using SPSS 18.0 for Windows (Chicago, IL, USA) and STATA 12 (College Station, TX, USA).

## Results

Demographic, clinical data, and laboratory results at baseline are shown in **Table 1**. The studied population was predominantly composed of middle-aged, non-black males on hemodialysis for eight years, and the main vascular access was an arteriovenous fistula. Hypertension and polycystic kidney disease were the most common CKD etiology. Comorbidities were frequent, and 34% of the patients had cardiovascular disease. Most patients (59%) had been prescribed erythropoietin, 25% acetylsalicylic acid, and none used warfarin or other direct oral anticoagulants. The mean heparin dose per hemodialysis session was 6643 UI, and in 56% of the patients, it was administered as a fractional dose. As expected, the ACT value increased at the 2^nd^ hour, returning to the baseline values at the 4^th^ hour. All demographic and clinical data between Groups 1 and 2 were similar, showing adequate randomization.

**Table 1:** Characteristics of the studied population.

	n=32	Group 1	Group 2	*p-value
		(n=16)	(n=16)	
**Men, n (%)**	19 (59)	11 (69)	8 (50)	0.28
**Age (years)**	52 ± 13	54 ± 13	50 ± 13	0.36
**Non-black, n (%)**	26 (81)	12 (75)	14 (87)	0.26
**CKD etiology, n (%)**				0.53
Undetermined	13 (41)	5 (31)	8 (50)	
Hypertension	5 (16)	4 (25)	1 (6)	
Polycystic Kidney Disease	5 (16)	2 (12)	3 (19)	
Diabetes	3 (9)	2 (12)	1 (6)	
Other	6 (19)	3 (19)	3 (19)	
**Time on hemodialysis (months)**	100 ± 32	114 ± 102	86 ± 85	0.40
**Arteriovenous fistula, n (%)**	23 (72)	14 (87)	9 (56)	0.10
**Hypertension, n (%)**	23 (72)	13 (81)	10 (62)	0.43
**Diabetes, n (%)**	4 (12)	3 (19)	1 (6)	0.60
**Cardiovascular disease, n (%)**	11 (34)	6 (37)	5 (31)	0.71
**Smokers, n (%)**	6 (19)	4 (25)	2 (12)	0.65
**Cancer, n (%)**	2 (6)	1 (6)	1 (6)	1.00
**Body mass index (Kg/m^2^)**	24.5 ± 2.8	23.7 ± 2.9	25.2 ± 2.6	0.12
**Erythropoietin use, n (%)**	19 (59)	9 (56)	10 (62)	0.72
**Acetylsalicylic acid use, n (%)**	8 (25)	4 (25)	4 (25)	1.00
**Unfractionated heparin dose/body weight (UI/kg)**	99.9 ± 12.0	101.6 ± 12.7	97.8 ± 11.3	0.43
**Unfractionated heparin administration, n (%)**				0.72
Bolus	14 (44)	6 (37)	8 (50)	
Fractioned	18 (56)	10 (62)	8 (50)	
**ACT baseline (pre-heparinization) (s)**	132 ± 56	136 ± 36	128 ± 72	0.70
**ACT 2^nd^ hour (s)**	195 ± 60	202 ± 66	190 ± 55	0.57
**ACT 4^th^ hour (s)**	128 ± 32	138 ± 33	118 ± 28	0.07

CKD, chronic kidney disease; ACT, activated clotting time; Group 1 (MCO followed by HF), Group 2 (HF followed by MCO); Entries are absolute and percentage frequencies and means ± Standard Deviations, *refers to the comparison between group 1 and group 2 using t-tests or the Mann-Whitney U-test, and chi-squared tests.

Considering the absence of a carryover effect on laboratory, ultrafiltration ratio, and blood pressure parameters, the data were regrouped according to the membrane type regardless of the study week (**Table 2**). There was no significant difference within groups during the study. An ANOVA model with repeated measures was built to compare the behavior of those parameters on MCO and High-flux groups, and no time, treatment, or interaction effects were observed.

**Table 2:** Laboratory tests in MCO and High-flux groups before and after 12 weeks of treatment with MCO/High-flux membranes.

	MCO group		High Flux Group		
The phase of each treatment	Initial	Final	Initial	Final	p MCO vs. High-flux
**Hemoglobin (g/dL)**	11.10 ± 1.66	11.58 ± 1.00	10.94 ± 1.22	11.19 ± 1.57	0.715
**Platelets (10^3^/mm^3^)**	177690 ± 44630	193929 ± 63307	175688 ± 61483	186300 ± 44939	0.597
**Glucose (mg/dL)**	88.10 ± 27.70	82.07 ± 2.11	94.09 ± 40.55	86.83 ± 28.86	0.742
**Glycosylated Hemoglobin(%)**	5.63 ± 0.86	5.85 ± 0.97	5.71 ± 0.84	5.72 ± 0.76	0.151
**Urea (mg/dL)**	146.32 ± 44.91	141.89 ± 31.35	151.75 ± 31.16	150.63 ± 43.41	0.484
**Creatinine (mg/dL)**	11.93 ± 2.09	11.53 ± 2.70	11.92 ± 2.48	11.56 ± 2.22	0.880
**Sodium (mEq/L)**	136.14 ± 2.61	136.29 ± 3.33	136.78 ± 3.01	135.83 ± 3.35	0.098
**Potassium (mEq/L)**	5.59 ± 0.97	5.77 ± 0.85	5.49 ± 0.97	5.76 ± 0.78	0.975
**Ionized calcium (mmol/L)**	1.17 ± 0.10	1.15 ± 0.11	1.17 ± 0.10	1.17 ± 0.10	0.332
**Phosphorus (mg/dL)**	5.02 ± 1.67	5.06 ± 1.72	4.79 ± 1.34	4.84 ± 1.41	0.671
**Bicarbonate(mmol/L)**	19.53 ± 2,87	20.04 ± 2.72	19.76 ± 2.55	19.91 ± 2.69	0.393
**Albumin (g/dL)**	3.93 ± 0.19	3.77 ± 0.21	3.86 ± 0.29	3.85 ± 0.34	0.098
**Total cholesterol (mg/dL)**	151.07 ± 40.89	149.14 ± 38.21	144.72 ± 30.62	147.33 ± 43.11	0.562
**Triglycerides (mg/dL)**	189.52 ± 103.54	184.61 ± 192.81	165.31 ± 94.10	156.87 ± 111.04	0.827
**Body mass index (kg/m^2^)**	24.65 ± 2.75	24.50 ± 3.00	24.16 ± 3.12	24.12 ± 3.08	0.328
**Von Willebrand factor (pg/mL)**	9882.18 ± 8452.29	11625.00 ± 8436.72	9723.96 ± 8921.21	10766.67 ± 18729.85	0.952
**C- reactive protein (mg/dL)**	0.57 ± 0.60	0.77 ± 1.38	0.56 ± 0.75	0.48 ± 0.62	0.383
**Tumoral Necrosis Factor α (pg/mL)**	32.50 ± 13.83	27.25 ± 9.91	33.89 ± 14.72	30.37 ± 11.42	0.378
**Interleukin 1ß (pg/mL)**	1.92 ± 5.52	0.96 ± 1.08	1.72 ± 4.29	1.01 ± 1.14	0.851
**Interleukin 6 (pg/mL)**	13.21 ± 41.10	12.74 ± 41.77	12.03 ± 38.92	17.48 ± 49.05	0.702
**Ultrafiltration ratio (L)**	2.55 ± 0.87	2.42 ± 0.88	2.55 ± 0.82	2.39 ± 0.88	0.653
**Systolic blood pressure (mmHg)**	113 ± 22	119 ± 20	115 ± 26	115 ± 28	0.283
**Diastolic blood pressure (mmHg)**	72 ± 14	76 ± 14	73 ± 17	73 ± 19	0.311

Comparison within the group (at the beginning and the end of the treatment MCO and High flux) was evaluated by two-sided t-test and Wilcoxon tests (* = p ≤0.05) and between groups by ANOVA model (p interaction values).

**Figure 2:**
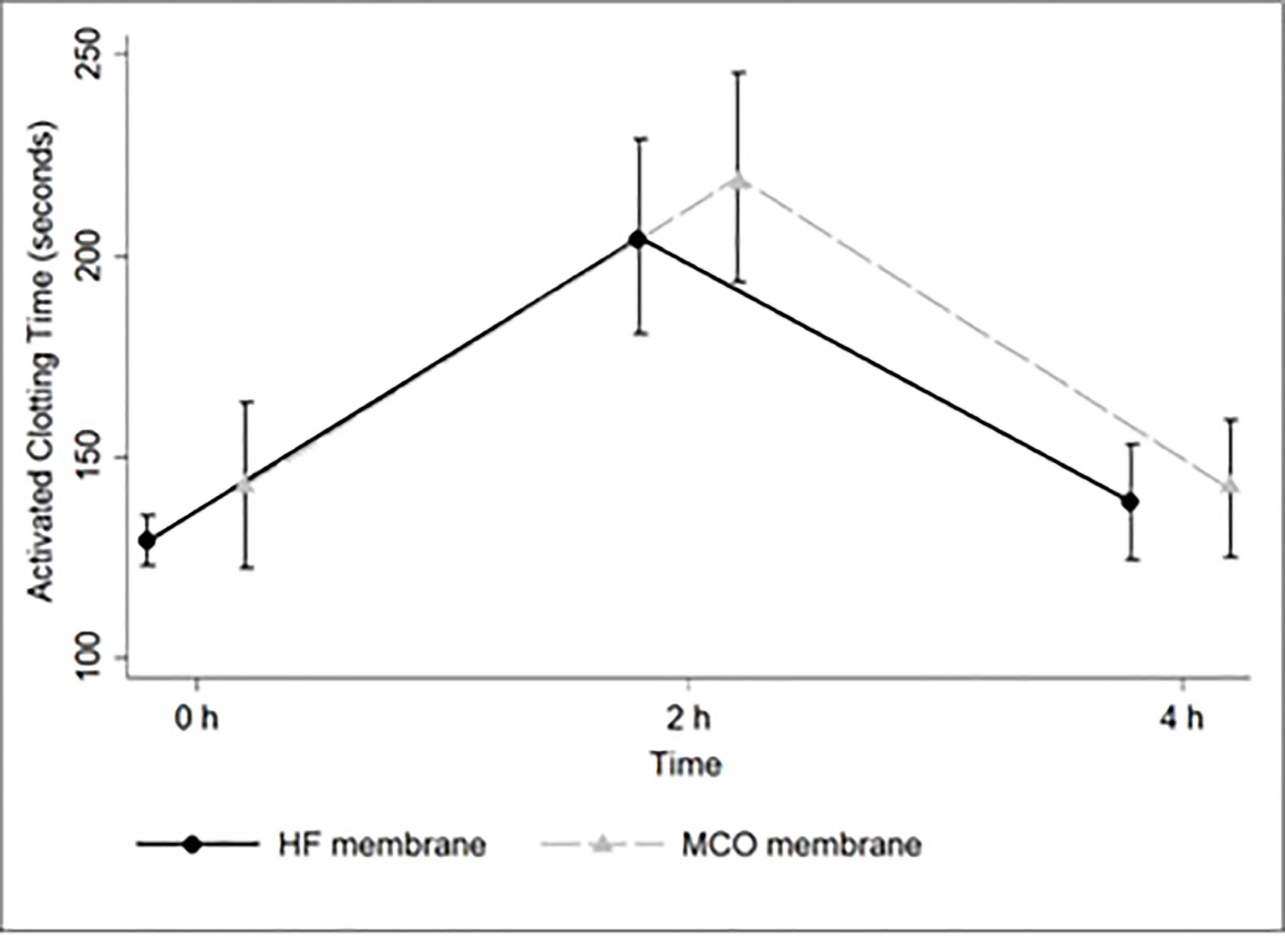
ACT curve throughout the dialysis session in the HF and MCO groups. MCO, medium cut-off membrane; HF, High Flux membrane.

There were no carryover effects on the ACT at pre-heparinization (p=0.334), 2^nd^ h (p=0.70), and 4^th^ h (p=0.45). Therefore, the population was regrouped according to the treatment, regardless of the study week. **Table 3** shows the ACT values at baseline (pre-heparinization), 2^nd^, and 4^th^ hour in the MCO and HF Groups. An ANOVA model for repeated measures showed a significant time effect but no treatment or interaction effects (**Figure 2**).

**Table 3:** ACT measurements during dialysis sessions before and after 12 weeks of treatment with MCO/High-flux membranes.

	MCO group	High flux group	p treatment	p time	p interaction
	**Mean ± SD**	**Mean ± SD**	0.29	<0.001	0.71
**ACT pre heparinization**	143 ± 54	129 ± 17			
**ACT 2^nd^ hour**	219 ± 68	205 ± 65			
**ACT 4^th^ hour**	142 ± 45	139 ± 38			

ACT= activated clotting time;

A similar result was observed when an adjusted ANOVA model was built. There was a time (p<0.001) but no treatment (p=0.25) or interaction (p=0.69) effects. The covariables used in this model included the use of erythropoietin (p=0.91) and acetylsalicylic acid (p=0.32) and levels of hemoglobin (p=0.50), platelets (p=0.40), vWf (p=0.58), CRP (p=0.35), TNFα (p=0.83), IL6 (p=0.94), IL1ß (p=0.99), and room temperature (p=0.26).

During the study, there were 4 patients withdrawn due to kidney transplant (n=1), changing dialysis center (n=2), and death (n=1). Considering the patients who completed the study (n=28), additional analysis with paired samples was performed. The ACT values remained similar between MCO and HF Groups, at baseline (143 ± 54 vs. 129 ± 17 sec, MCO vs. HF, respectively, p= 0.20), at 2^nd^ hour (219 ± 68 vs. 201 ± 62 sec, p=0.30) and 4^th^ hour (142 ± 45 vs138 ± 39 sec, p=0.76).

Finally, there were no bleeding events or clotting of the extracorporeal system in the groups throughout the study.

## Discussion/Conclusion

This study showed no difference between the effect of MCO or HF membranes on the ACT test during hemodialysis.

The interaction between blood and the extracorporeal circuit on hemodialysis, mainly the dialysis membrane, activates the complement system and the coagulation cascade, requiring anticoagulants during the procedure (6, 18). The coagulation activation by the dialyzer is partially dependent on its biocompatibility characteristics. The switch of the former cellulosic membranes to the more biocompatible synthetic membranes, currently used on chronic hemodialysis, has decreased inflammation and the activation of the complement system, leading to a decreased hypercoagulable state (11). MCO dialyzer membranes could activate coagulation at a minor level by providing a better clearance of the middle molecules as inflammatory cytokines. However, a recent study using an *in vitro* model demonstrated that the activity of coagulation factors and their inhibitors remains unaltered with MCO membranes (16, 19). In the present study, we could not show differences in the ACT curves between both HF and MCO membranes, regardless of the adjustment to CRP, IL6, TNFα, IL1ß, and vWf. There was also no significant difference in inflammatory markers found in HF and MCO groups, which can be inconsistent with studies that show that MCO membranes reduce inflammation (16). This might be due to the population included in the study, as the presence of active inflammation was one of the exclusion criteria.

Moreover, the bigger pore size of MCO membranes compared to HF could be a concern regarding the heparinization scheme (20). Firstly, this physical characteristic of that membrane leads to a higher ultrafiltration rate and, consequently, to hemoconcentration inside the dialyzer (21). It is well known that the initial blood coagulation reaction could be accelerated under a hemoconcentration state (22). Interestingly, patients undergoing post-dilution hemodiafiltration that favors hemoconcentration within the dialyzer may need to increase the heparin dose (23). Secondly, the bigger pore size allows some loss of middle molecules, like albumin, which weighs 68000 Da (24). Considering that heparin’s molecular weight ranges from 3000 to 30000 Da, one could consider that heparin loss occurs during hemodialysis with the MCO membrane (25). Those two conditions could increase blood coagulability, requiring higher doses of heparin. However, we observed no difference in the ACT values with the use of MCO compared to HF membranes, despite the heparin dose being maintained unchanged during the study.

The ACT test was used in this study as a measurement method to evaluate the coagulation pattern (8). Although this test has been considered a point of care for monitoring anticoagulation during dialysis, some factors, such as platelet activation, room temperature, and hypothermia, could interfere with the reliability of its results (26, 27). In the present study, room temperature, hypothermia, and the number of platelets were controlled and included as covariables in the adjusted model.

One limitation of this study is the relatively small sample size, but the crossover and randomized design might strengthen it. To the best of our knowledge, this was the first study evaluating the MCO membrane coagulation activation pattern during hemodialysis.

In conclusion, there was no difference regarding the ACT test during dialysis therapy using HF or MCO membranes. This data suggests that no adjustment in the dose or administration method of heparin is necessary with the use of MCO dialysis membranes.

## Data availability statement

The original contributions presented in the study are included in the article/supplementary material. Further inquiries can be directed to the corresponding author.

## Ethics statement

The studies involving human participants were reviewed and approved by Ethics Committee of Federal University of São Paulo (n. 026386/2019). The patients/participants provided their written informed consent to participate in this study.

## Author contributions

Conception and design of the study: MC and SM. Generation, collection, analysis, and interpretation of data: IP, RA, MR, TG, LS and RW. Drafting and revision of the manuscript: IP, RA, AC and MC. Approval of the final version of the manuscript: IP, RA, AC and MC. All authors contributed to the article and approved the submitted version.
